# A Novel Transvaginal Cervical Cerclage Model for Resident Training

**DOI:** 10.15766/mep_2374-8265.11102

**Published:** 2021-03-02

**Authors:** Arlin Delgado, Rachael Cleberg, Alexis C. Gimovsky

**Affiliations:** 1 Obstetrics and Gynecology Resident, Department of Obstetrics and Gynecology, University of South Florida Morsani College of Medicine; 2 Physician, Capital Women's Care, Division 44; 3 Assistant Professor, Division of Maternal-Fetal Medicine, Department of Obstetrics and Gynecology, Women & Infants Hospital, Warren Alpert Medical School of Brown University

**Keywords:** Transvaginal Cervical Cerclage, Resident Teaching, OB/GYN, Maternal-Fetal Medicine, Perinatology

## Abstract

**Introduction:**

Vaginal suturing can be challenging to teach and learn due to the surgical assistant's limited operative field visualization. Data on resident training and comfort with cerclage placement using models are limited. The aim of this activity was to assess learner satisfaction with practice using a novel model allowing for full visualization during transvaginal cervical cerclage placement.

**Methods:**

OB/GYN residents participated in a 1-hour combined lecture and hands-on cerclage training simulation with the novel model. Pre- and postsession survey responses were assessed with descriptive statistics and paired *t* tests.

**Results:**

Twenty residents with a median of 2 (*SD* = 1.6) years of residency experience participated. Ninety-five percent reported no prior cerclage simulation training; 60% reported placing cerclages in practice. Pre- and posttest analysis indicated a significant decrease in perceived need for further training (*M* = 4.05, *SD* = 1.07, vs. *M* = 3.45, *SD* = 0.86; *p* = .024) and an increase in comfort performing a cerclage placement (*M* = 2.55, *SD* = 1.16, vs. *M* = 3.85, *SD* = 0.79; *p* < .001). After the simulation, residents reported more comfort in cerclage placement with decreasing supervision (*M* = 2.05, *SD* = 1.02, vs. *M* = 2.30, *SD* = 1.01; *p* = .021); 90% reported that learning to place a cerclage was easy.

**Discussion:**

Implementing a novel, low-cost model allowing full operative field visualization significantly improved reported comfort regarding cervical cerclage placement and resulted in high satisfaction amongst residents. Future research should evaluate the training's impact on clinical skills.

## Educational Objectives

By the end of this activity, learners will be able to:

1. Define cervical insufficiency.
2. Understand the instruments needed to perform a cerclage.
3. Perform the procedural steps to place a McDonald cervical cerclage.
4. Understand the complications associated with cerclage placement.

## Introduction

Cervical insufficiency is associated with preterm birth, and current therapeutic options are usually limited to progesterone and cerclage placement.^1^ Current American College of Obstetricians and Gynecologists guidelines recommend the placement of cervical cerclage if a woman has a history of multiple preterm losses secondary to cervical insufficiency, if a woman has a history of preterm birth and has a short cervix as measured on transvaginal ultrasound of less than 2.5 cm, or if a woman has advanced cervical dilation prior to viability.^2^

Cerclage placement requires a health care provider to be technically skilled in vaginal suturing. The Accreditation Council for Graduate Medical Education (ACGME) Milestones list performance of cervical cerclage as a Level 4 Milestone under Obstetrical Technical Skills.^3^ As a result, there is an emphasis on proper training for residents. Yet this skill can be challenging to teach and to learn due to limited operative field visualization by the learner and the teacher. Only two prior do-it-yourself cervical cerclage training models have been reported for training purposes. The first model utilizes multiple components, including frozen cow muscle, and is technically complex.^4^ The existing model provides a low-cost option but is geared towards maternal-fetal medicine fellows or attendings performing cerclage in the setting of a prolapsing amniotic sac.^5^

The use of simulation training amongst residents positively impacts both their ability and confidence to perform important procedures, such as cervical exams, shoulder dystocia, vaginal tape placement, and robotic surgical skills.^6–9^ Due to the limited number of tools for resident training in cerclage placement using homemade/do-it-yourself models^4,5^ and the absence of commercially available models for these training purposes, we constructed a low-cost model to assist residents in their training. The aim of this scholarly project was to assess the use of a novel transvaginal cervical cerclage model for resident training.

## Methods

### Model Technique

Using a clear 1-L bottle, 0-vicryl suture, and a hair-bun maker (a rolled mesh donut purchased from the hair care aisle at a local convenience store), the model was created in several easy steps outlined in Appendix A. This model also allowed one to create varying vaginal canal lengths and cervix firmness and sizes based on the materials chosen.

### Training Session and Survey

First, an approximately 20- to 30-minute lecture on cerclages in the context of preterm birth was given via PowerPoint presentation (Appendix B). The presentation was adapted from the Society of Maternal-Fetal Medicine fellow lecture produced by Vincenzo Berghella.^10^ This was followed by 40 minutes of hands-on training. No prior knowledge or experience was necessary. To facilitate appropriate and timely feedback, junior and senior residents were paired up and distributed among four models, and two to three maternal-fetal medicine faculty members circulated amongst the groups to provide feedback as well.

Residents participated in a pre- and postsession survey. After the presession survey had been completed, the cerclage model was demonstrated as follows:

1. The suture was first inserted at 12 o'clock, exiting at approximately the 9 o'clock position, continuing in a counterclockwise manner so as to have four bites ending back at 12 o'clock (for a right-handed surgeon, but in reverse for a left-handed surgeon).
2. The suture was taken at both ends, and a firm surgical knot was tied.

Once the model had been demonstrated, each resident performed at least one cerclage on it. Lastly, a postsession survey was administered.

The presession survey included the following resident self-reported demographic data: residency year, prior cerclage placement, and number performed. Pre- and postsession surveys, found in Appendix C, asked residents' views regarding comfort placing a cerclage and need for further training, using a 5-point Likert scale (1 = *Strongly Disagree,* 5 = *Strongly Agree*). Perceived need for supervision was assessed using the ACGME resident evaluation scale.^3^ This scale was used throughout residency training to assess resident skills and growth.

OB/GYN residents at George Washington University completed this activity in a 1-hour educational training session on transvaginal cervical cerclage placement. This scholarly project was approved by the George Washington University Institutional Review Board.

All responses were analyzed using Microsoft Excel 2013. Continuous variables were assessed using two-tailed paired *t* tests, and categorical variables were assessed using chi-squares or the Fisher exact test.

## Results

A total of 20 residents with a median of 2 (*SD* = 1.6) years of residency experience participated. Additional demographic and experiential data are reported in Table 1.

**Table 1.**
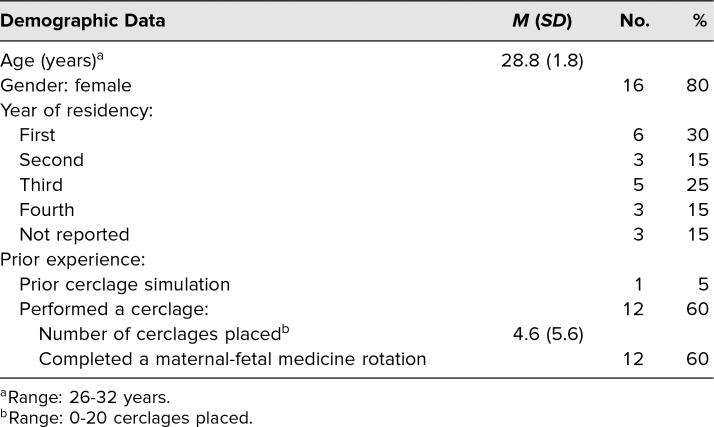
Demographics of Participants (*n* = 20)

Compared to presession scores, postsession analysis showed a decrease in the perception of need for further training after cerclage simulation (pre: *M* = 4.05, *SD* = 1.07, vs. post: *M* = 3.45, *SD* = 0.86; *p* = .024). Ninety percent of residents reported that learning to place a cerclage was easy after the simulation, versus 45% beforehand. Residents also reported a significant increase in comfort performing a cerclage placement compared to presimulation (pre: *M* = 2.55, *SD* = 1.16, vs. post: *M* = 3.85, *SD* = 0.79; *p* < .001). Residents specified more comfort in performing a cerclage in the context of decreasing supervision in the posttest survey in comparison to the pretest survey (pre: *M* = 2.05, *SD* = 1.02, vs. post: *M* = 2.30, *SD* = 1.01; *p* = .021; see the Figure). Seventy percent of residents strongly agreed this simulation training was helpful, and no residents disagreed. All residents agreed or strongly agreed that program directors should recommend this training tool.

**Figure.**
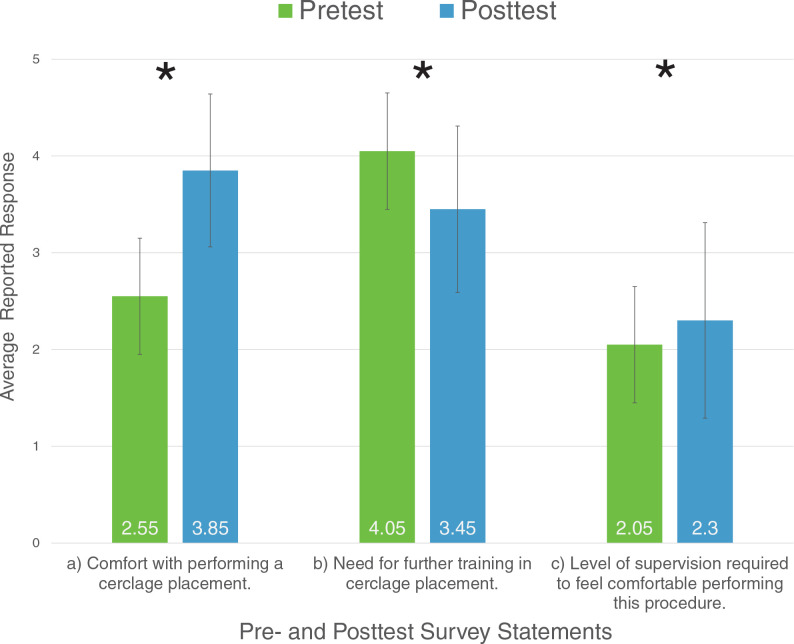
A comparison of resident-reported views pre- and postsimulation training with the cervical cerclage model. Statements regarding views are reported along the horizontal access, and responses on 5-point Likert scales are on the vertical axis. In statement c, an increasing score reflects increasing comfort with decreasing supervision. Asterisk indicates statistical significance (*p* < .05). Error bars indicate standard positive and negative error. Statement a key: 1 = *Very Uncomfortable,* 2 = *Uncomfortable,* 3 = *Neutral,* 4 = *Comfortable,* 5 = *Very Comfortable.* Statement b key: 1 = *Strongly Disagree,* 2 = *Disagree,* 3 = *Neutral,* 4 = *Agree,* 5 = *Strongly Agree.* Statement c key: 1 = *Requires Supervision,* 2 = *Frequently Requires Supervision,* 3 = *Occasionally Requires Supervision,* 4 = *Ready for Unsupervised Practice,* 5 = *Role Model.*

In comparing the responses of residents with experience in cerclage placement to those without, we found that the training resulted in larger gains in comfort for residents without experience (Table 2).

**Table 2.**
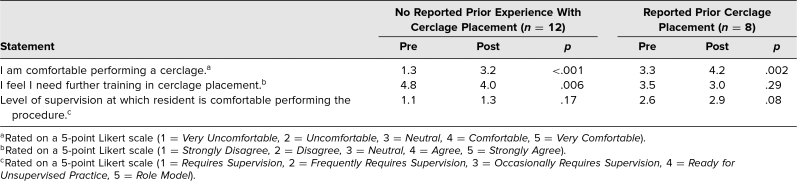
Pre- and Posttraining Average Responses by Prior Reported History of Cerclage Placement

## Discussion

We developed a teaching activity for OB/GYN residents to learn cervical cerclage placement using a novel model. Cervical cerclage placement can be a skill that is challenging to teach and learn due to barriers experienced in the operating room but is also a critical skill OB/GYN residents must learn for proper standard of care in patients with cervical insufficiency with an indication and desire for cerclage placement.

Reflecting on this educational activity, we saw that it significantly increased resident comfort regarding transvaginal cerclage placement, especially among residents with no prior history of cerclage placement. The model utilized in the activity provides a low-cost option with the ability to vary cervical and vaginal anatomy. Another benefit of this model is that the bottle that simulates the vaginal canal can be clear, enabling maximum visualization for learners and teachers, or can be made opaque (i.e., painted or covered externally with paper or cloth) to make the simulation more challenging as residents' skills increase with ongoing practice during the activity. Overall, residents showed a high level of satisfaction with the activity as well.

Yet this training session could be improved in several ways. First, mounting the model, so as not to require one individual to hold the model still while another is practicing the procedure, would permit all learners to practice simultaneously. Additionally, increasing the number of models available would decrease the size of the groups around a model and increase hands-on experience. Due to the need for immediate feedback, pairing a senior resident with prior experience and a junior resident at the start of the session would decrease dependence on faculty for feedback and facilitate discussion on technique in real time.

The generalizability of this activity is limited because we have not tested whether the reported increase in comfort and satisfaction with using the model for practice translates to improved ability to perform clinical procedures. While the model utilized in this activity can be altered to emphasize differing size or widths of vaginal canals, its materials (e.g., the hair bun) do not properly illustrate the properties of the cervical and vaginal tissue when being manipulated by a surgeon for cerclage placement. This difference could negatively impact residents' ability to perform the procedure successfully in clinical practice.

Although we saw a change in the reported degree of need for further training and supervision, the residents' ongoing need highlights the importance of further didactic opportunities for learning cerclage placement. Our next step is to utilize a skills checklist (Appendix D) in a teach-the-teacher session prior to a second general didactic session. This should eliminate concerns that senior residents may not be competent in the skills themselves. This checklist will also be distributed to learners for use during the activity, as well as to guide constructive feedback. Future implementation should also focus on learner achievement of competency in both the simulated setting and the clinical setting. This could be evaluated by tracking clinical procedures performed by residents, utilizing the skills checklist at the time of those procedures. This direct feedback could then be directly compared to feedback previously received during the training session itself.

## Appendices

Cerclage Model Building Steps.docxAdapted Cervical Insufficiency Slide Deck.pptxPre- and Postsurvey.docxSkills Checklist.docx
All appendices are peer reviewed as integral parts of the Original Publication.
