# Supplementary material for: A Novel Transvaginal Cervical Cerclage Model for Resident Training
Source: MedEdPORTAL. 2021 Mar 2;17:11102. doi: 10.15766/mep_2374-8265.11102 (PMC7970640; doi:10.15766/mep_2374-8265.11102)
Supplement: Supplementary file 1 — Cerclage Model Building Steps.docxAdapted Cervical Insufficiency Slide Deck.pptxPre- and Postsurvey.docxSkills Checklist.docx [file mep_2374-8265.11102-s001.zip › A. Cerclage Model Building Steps.docx]

## Appendices

**Appendix A: Cerclage Model Building Steps**

-
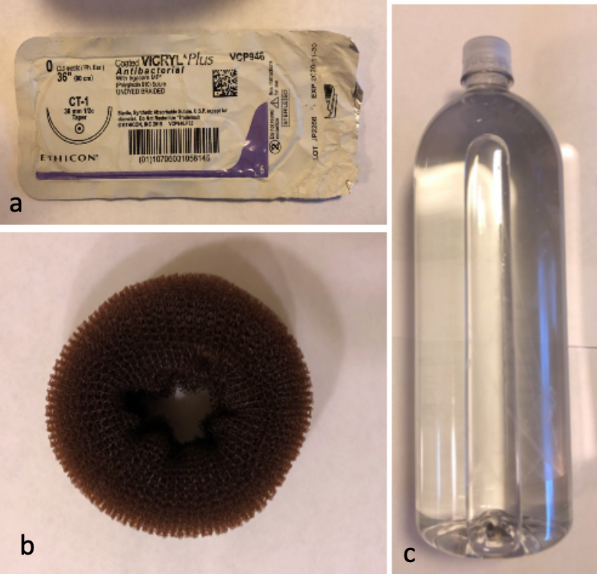
*Step 1:*  First, gather the following Cerclage Model Supplies depicted in (1a) that include: 0-vicryl suture used to suture pieces together, (1b) a hair bun maker used to create cervix and (1c) a 1-liter water bottle used to create vaginal canal.

1a

1c

1b

- *Step 2:* Next, cut the 1- liter water bottle, and follow the dashed lines depicted in (2a) representing where instructors should cut to create the necessary pieces depicted in (2b). Parts A and D of the water bottle should be discarded. Cut part B can vary depending on the desired length of the “vaginal canal”, by adjusting the cuts between parts B and C or parts A and B.


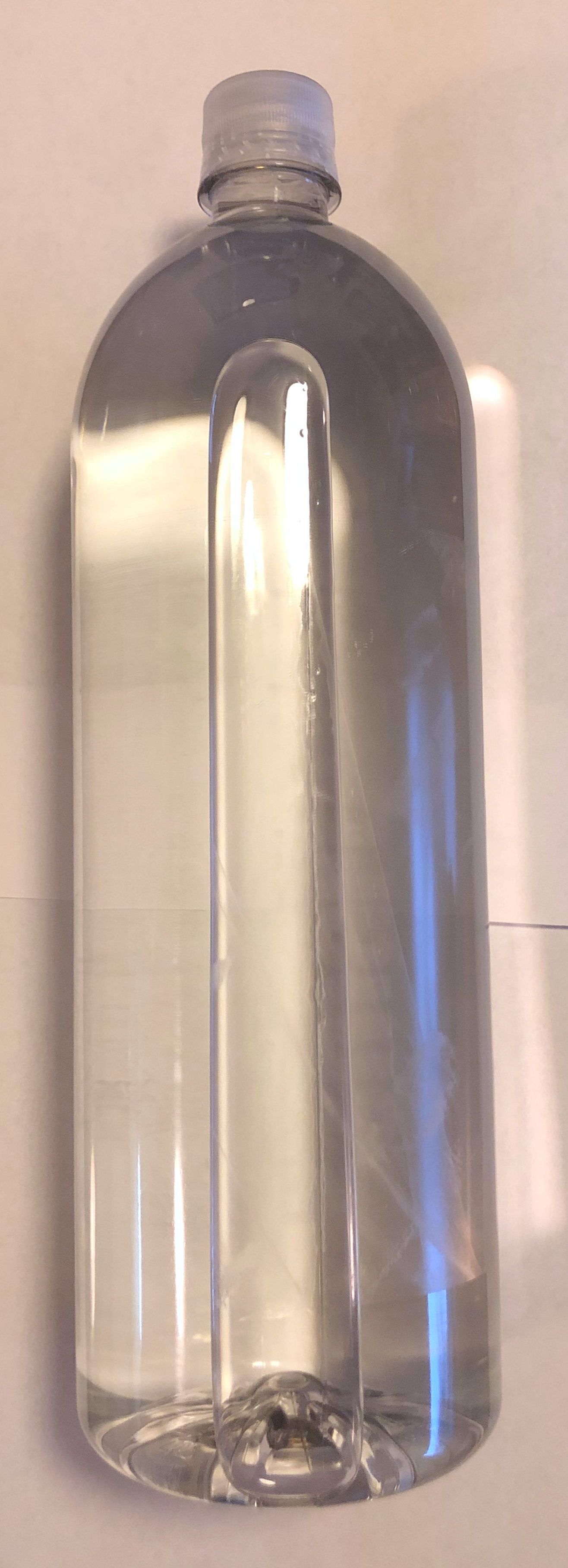


A

B
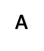


D

C

B

2a


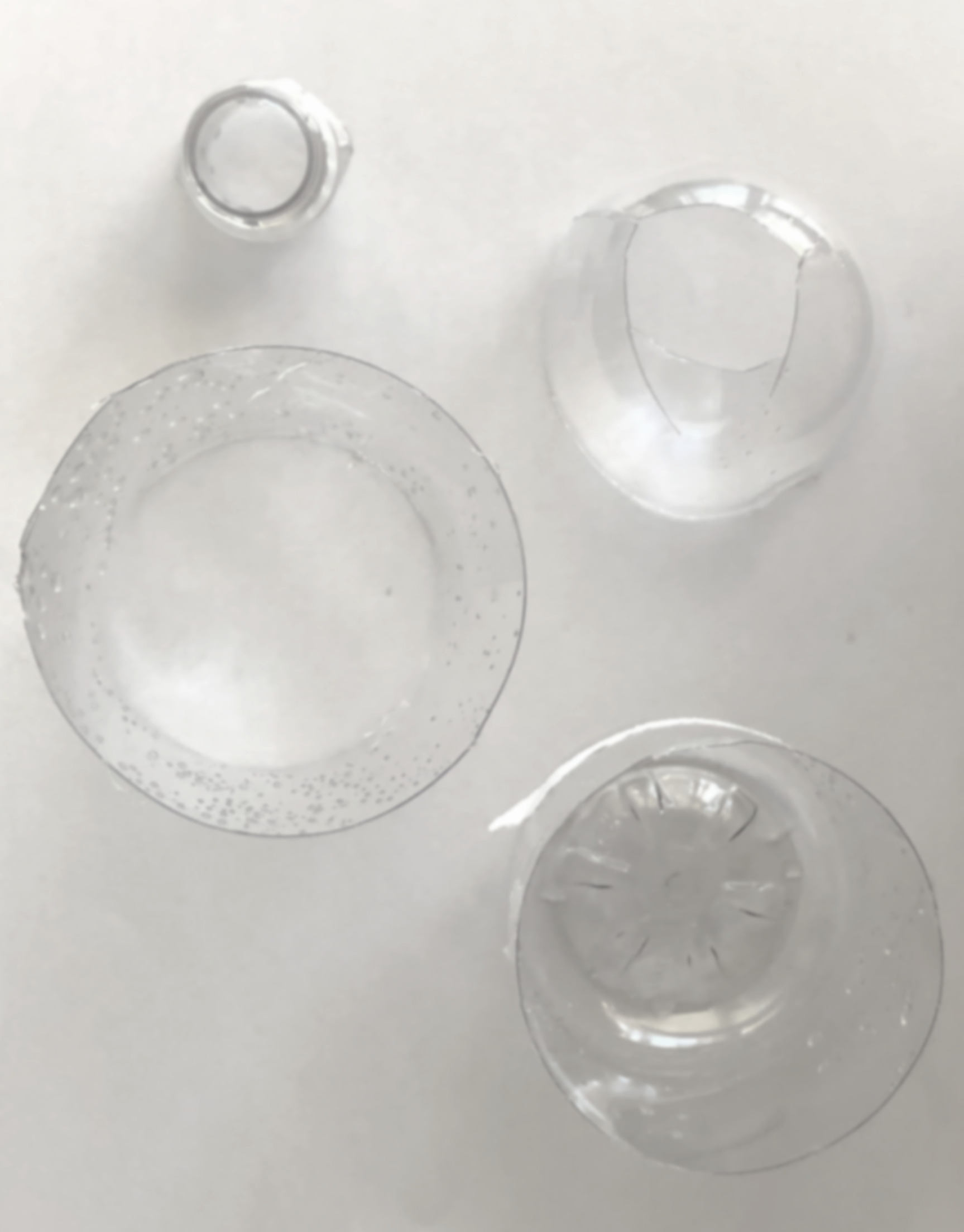


2b

A

B

C

D

*Step 3:* After cutting the water bottle as above in Step 2, take part C and locate the hair bun maker, that is chosen to make the “cervix” of model. The hair bun maker can also vary in size as too change the difficulty level of the model. As depicted below in (3b), place the hair bun maker on part C. The circles shown depict where to place 0-vicryl suture to properly attach the hair bun maker to part C. Lastly, take part C with hair bun maker and insert into part B, the “vaginal canal,” as depicted below in (3c). The circles in (3c) indicated points at which the 0-vicryl suture should be once again used to attach parts B and C together.


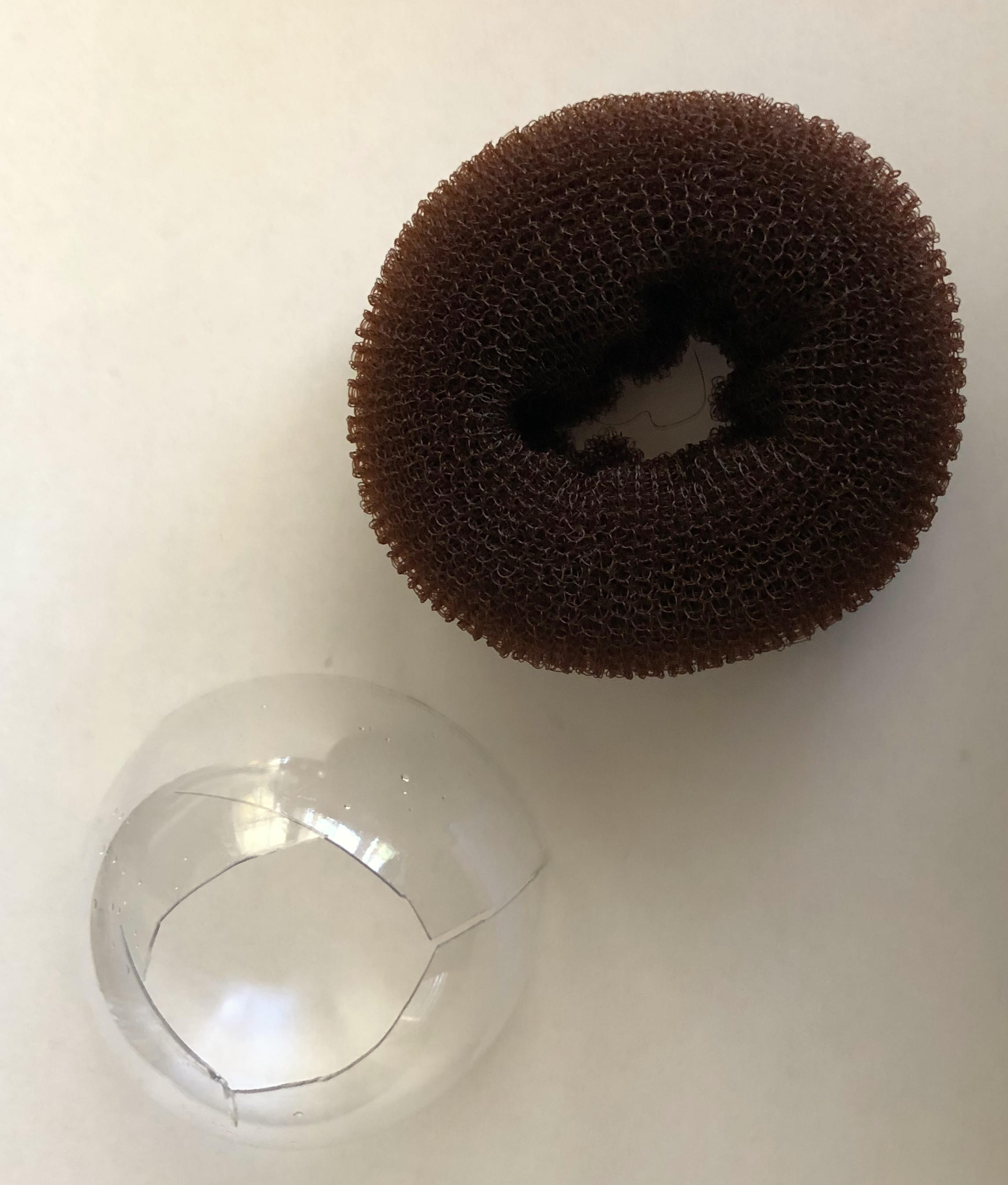

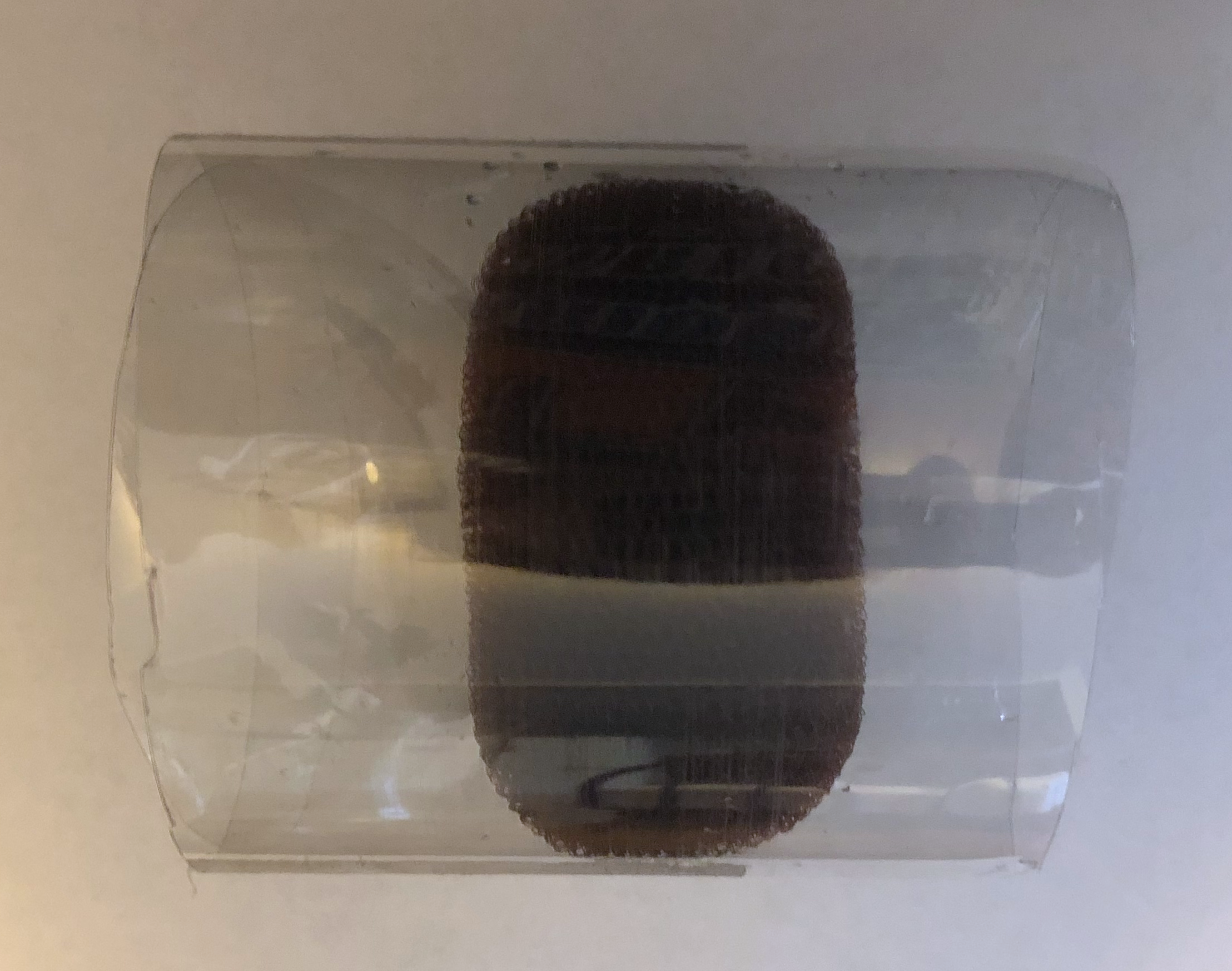

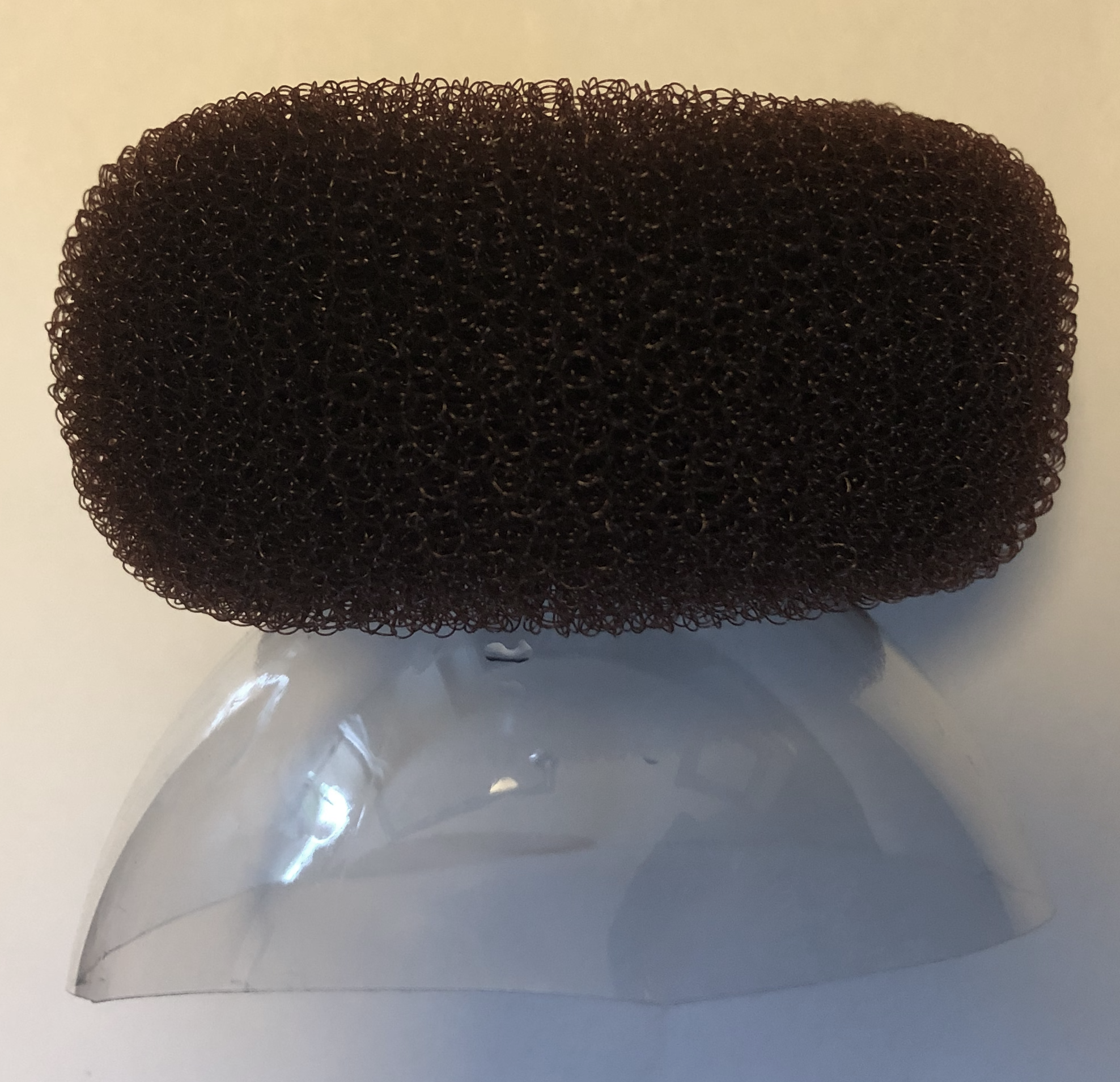


B

C

3b

3c

3a

C

*Step 4:* This is the final product of the Transvaginal Cerclage Model. In (4a) there is a model with large cervix and longer vaginal canal, and in (4b) model with smaller cervix in shorter vaginal canal.


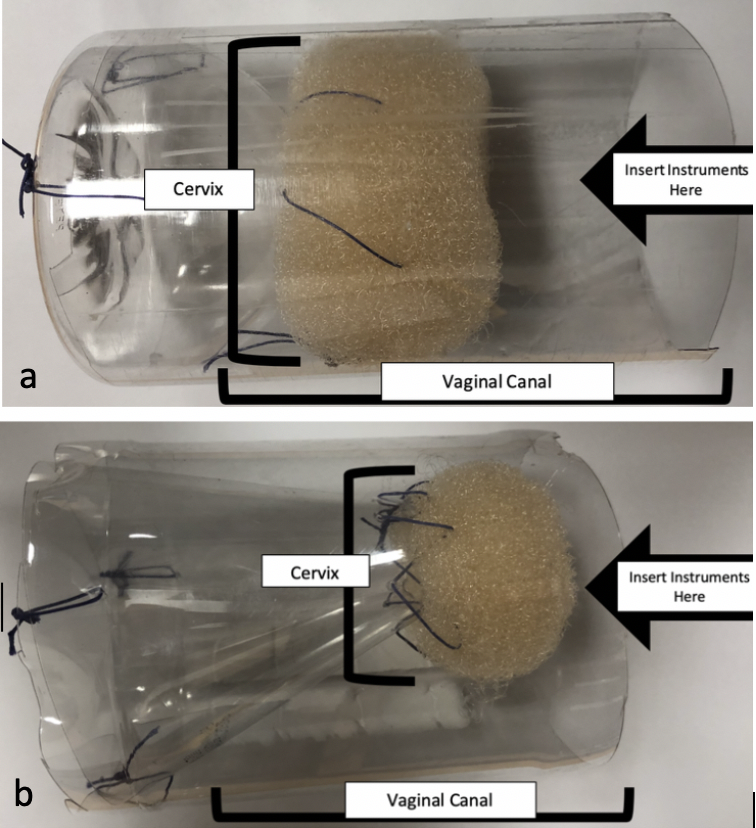


4

4

**References:**

All images and figures are Author Owned.
