# Supplementary material for: A Novel Transvaginal Cervical Cerclage Model for Resident Training
Source: MedEdPORTAL. 2021 Mar 2;17:11102. doi: 10.15766/mep_2374-8265.11102 (PMC7970640; doi:10.15766/mep_2374-8265.11102)
Supplement: Supplementary file 1 — Cerclage Model Building Steps.docxAdapted Cervical Insufficiency Slide Deck.pptxPre- and Postsurvey.docxSkills Checklist.docx [file mep_2374-8265.11102-s001.zip › B. Adapted Cervical Insufficiency Slide Deck.pptx]

## Slide 1
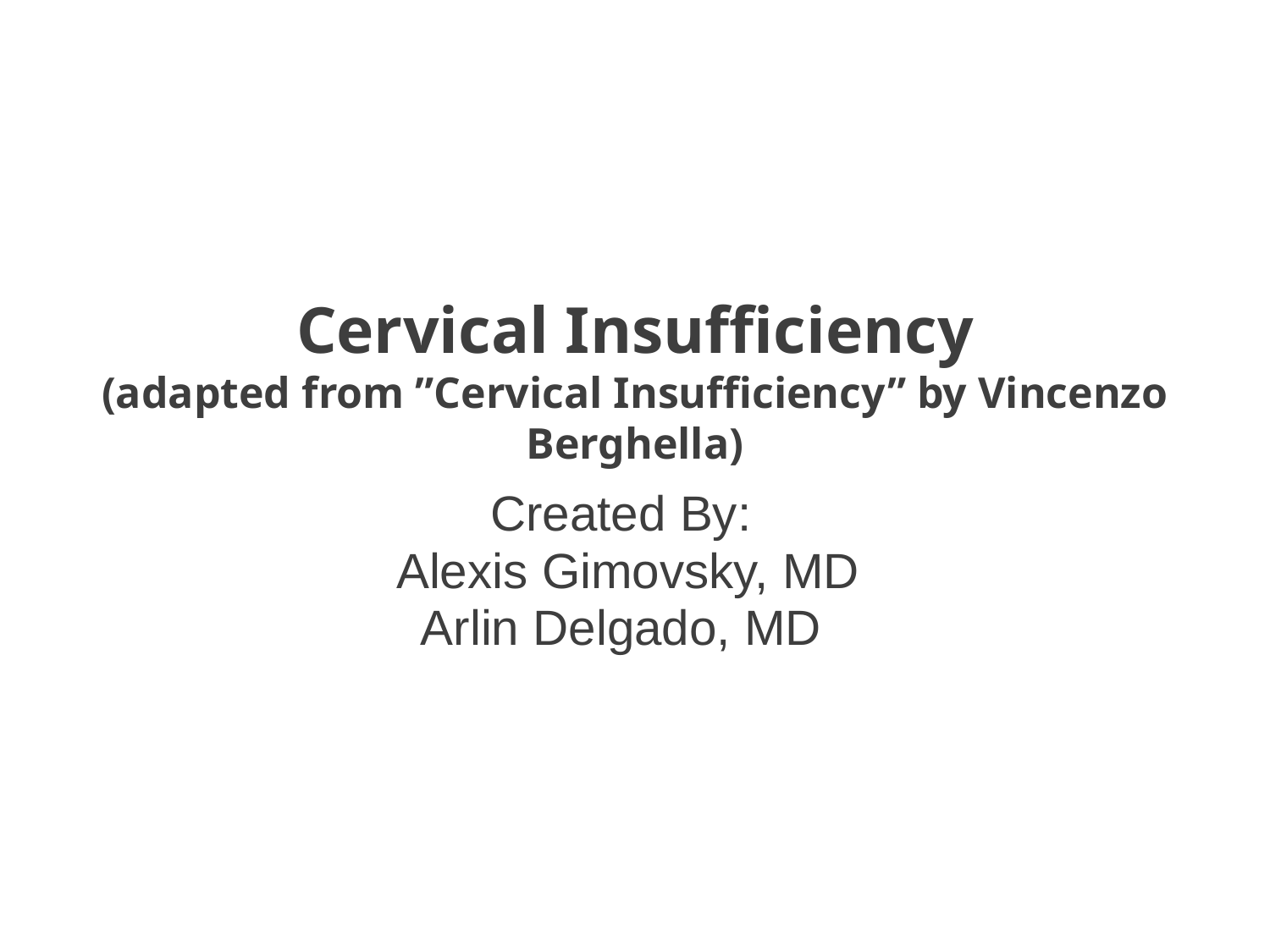

# Cervical Insufficiency (adapted from ”Cervical Insufficiency” by Vincenzo Berghella)
Created By:
Alexis Gimovsky, MD
Arlin Delgado, MD

## Slide 2
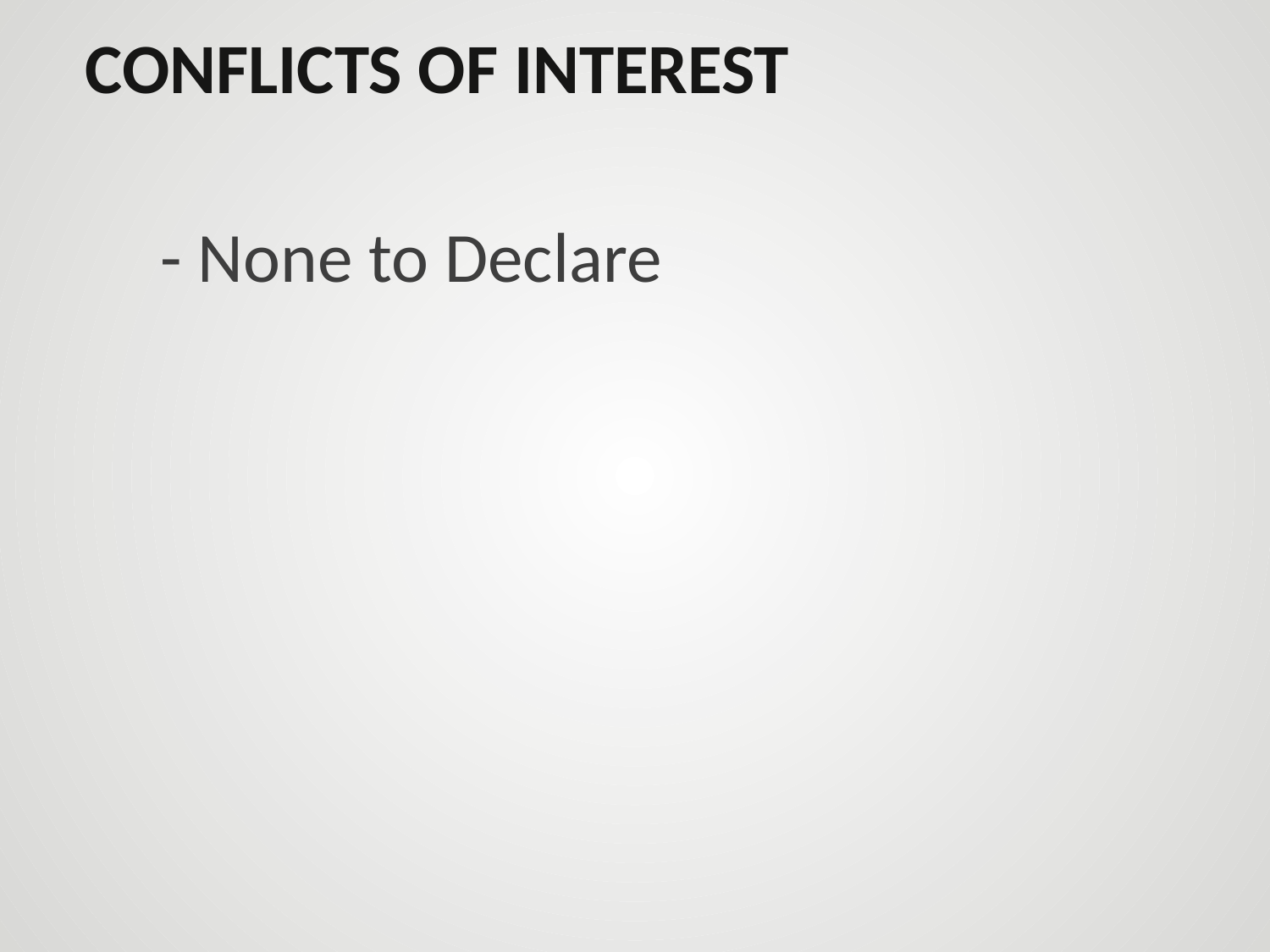

# Conflicts of Interest
- None to Declare

## Slide 3
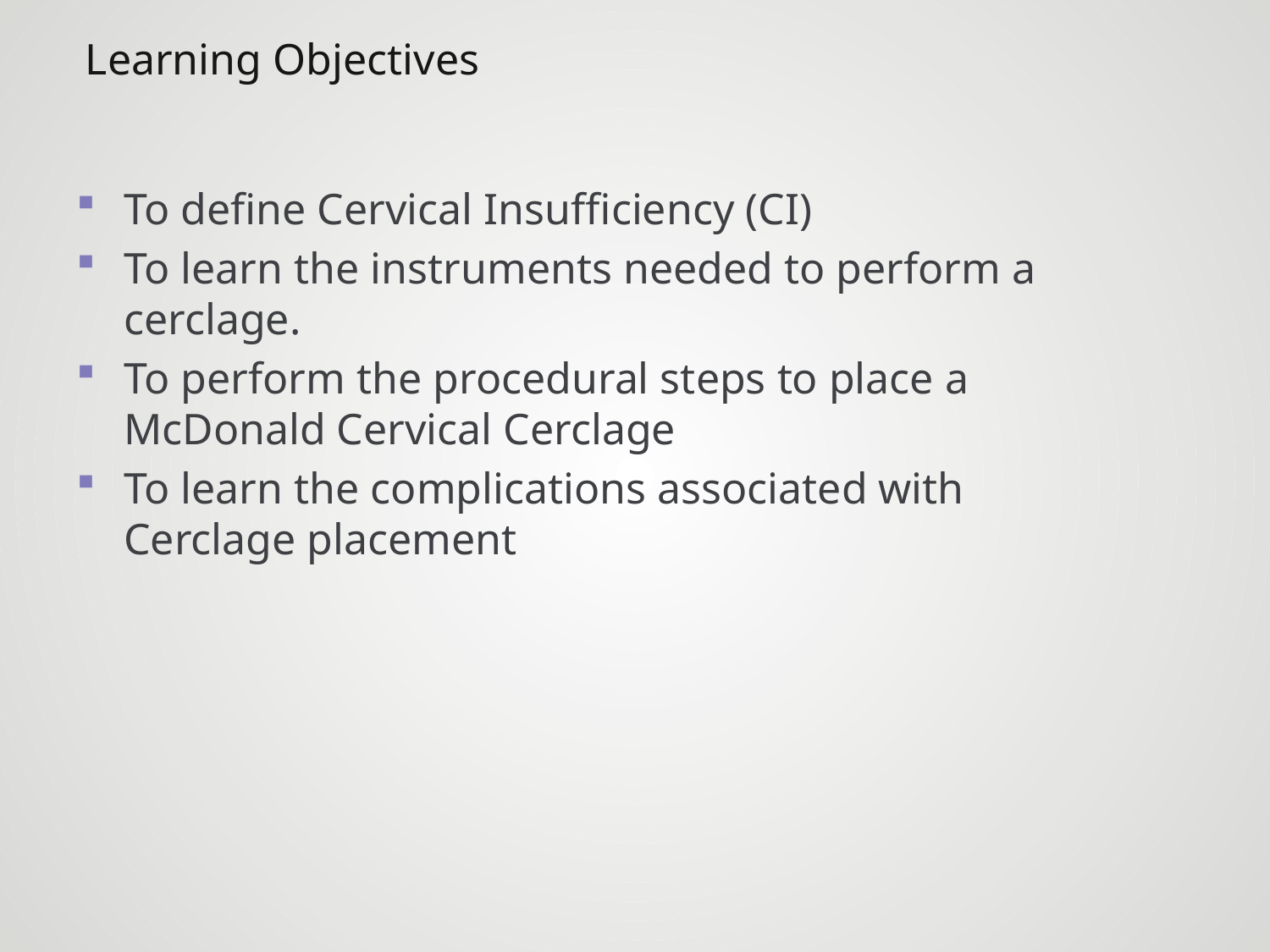

# Learning Objectives
To define Cervical Insufficiency (CI)
To learn the instruments needed to perform a cerclage.
To perform the procedural steps to place a McDonald Cervical Cerclage
To learn the complications associated with Cerclage placement

## Slide 4
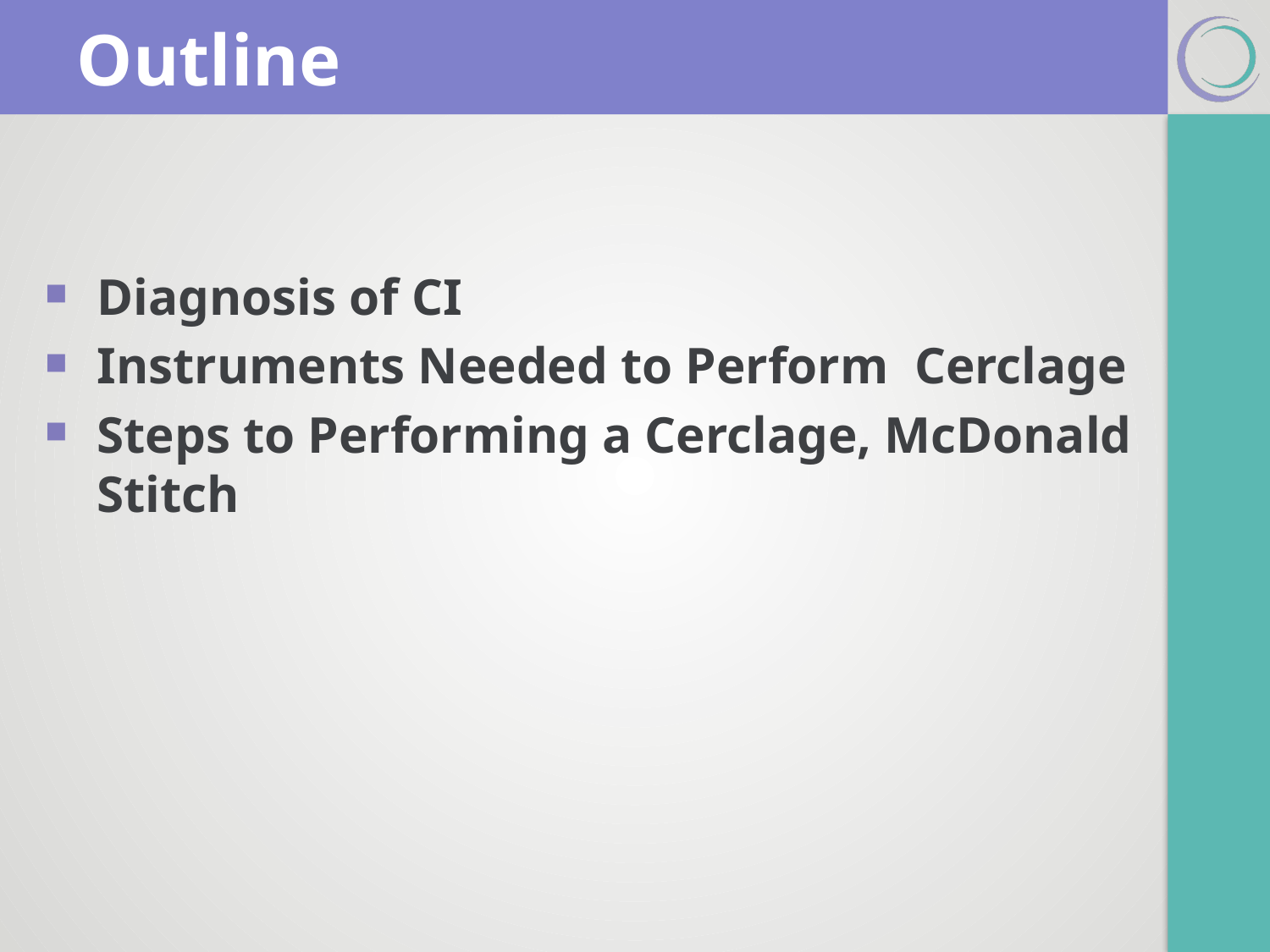

# Outline
Diagnosis of CI
Instruments Needed to Perform Cerclage
Steps to Performing a Cerclage, McDonald Stitch

## Slide 5
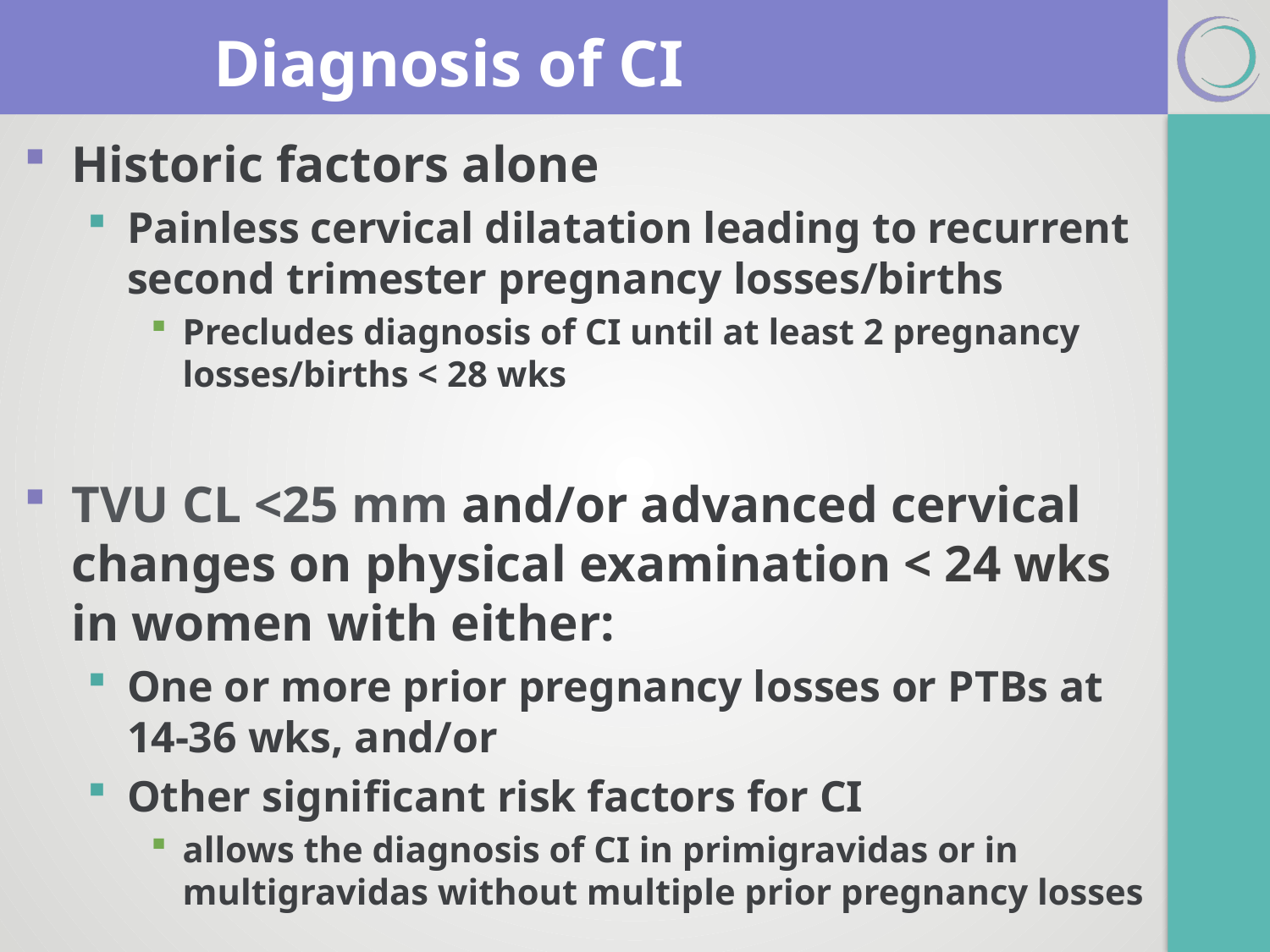

# Diagnosis of CI
Historic factors alone
Painless cervical dilatation leading to recurrent second trimester pregnancy losses/births
Precludes diagnosis of CI until at least 2 pregnancy losses/births < 28 wks
TVU CL <25 mm and/or advanced cervical changes on physical examination < 24 wks in women with either:
One or more prior pregnancy losses or PTBs at 14-36 wks, and/or
Other significant risk factors for CI
allows the diagnosis of CI in primigravidas or in multigravidas without multiple prior pregnancy losses

## Slide 6
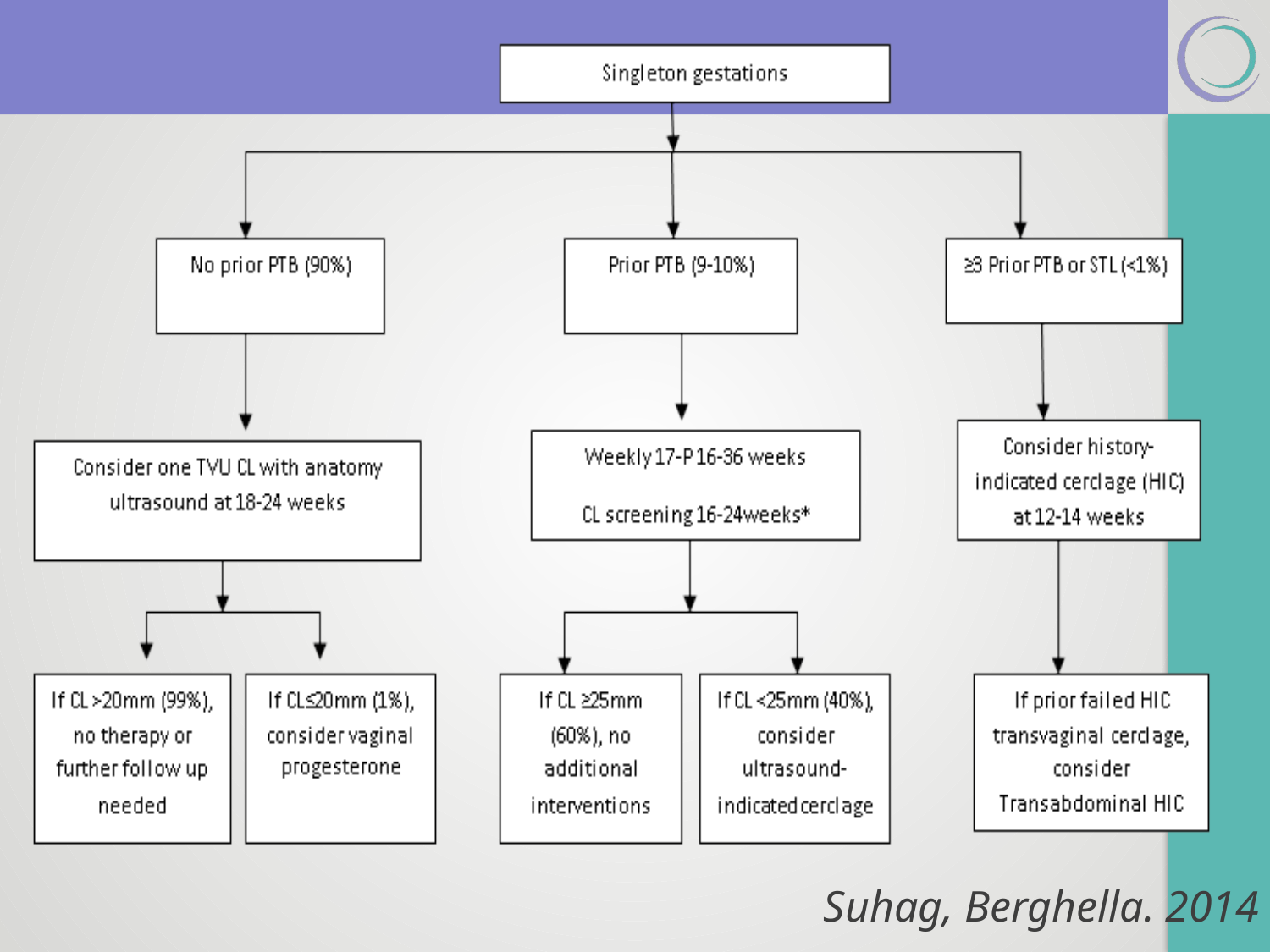

Suhag, Berghella. 2014

## Slide 7
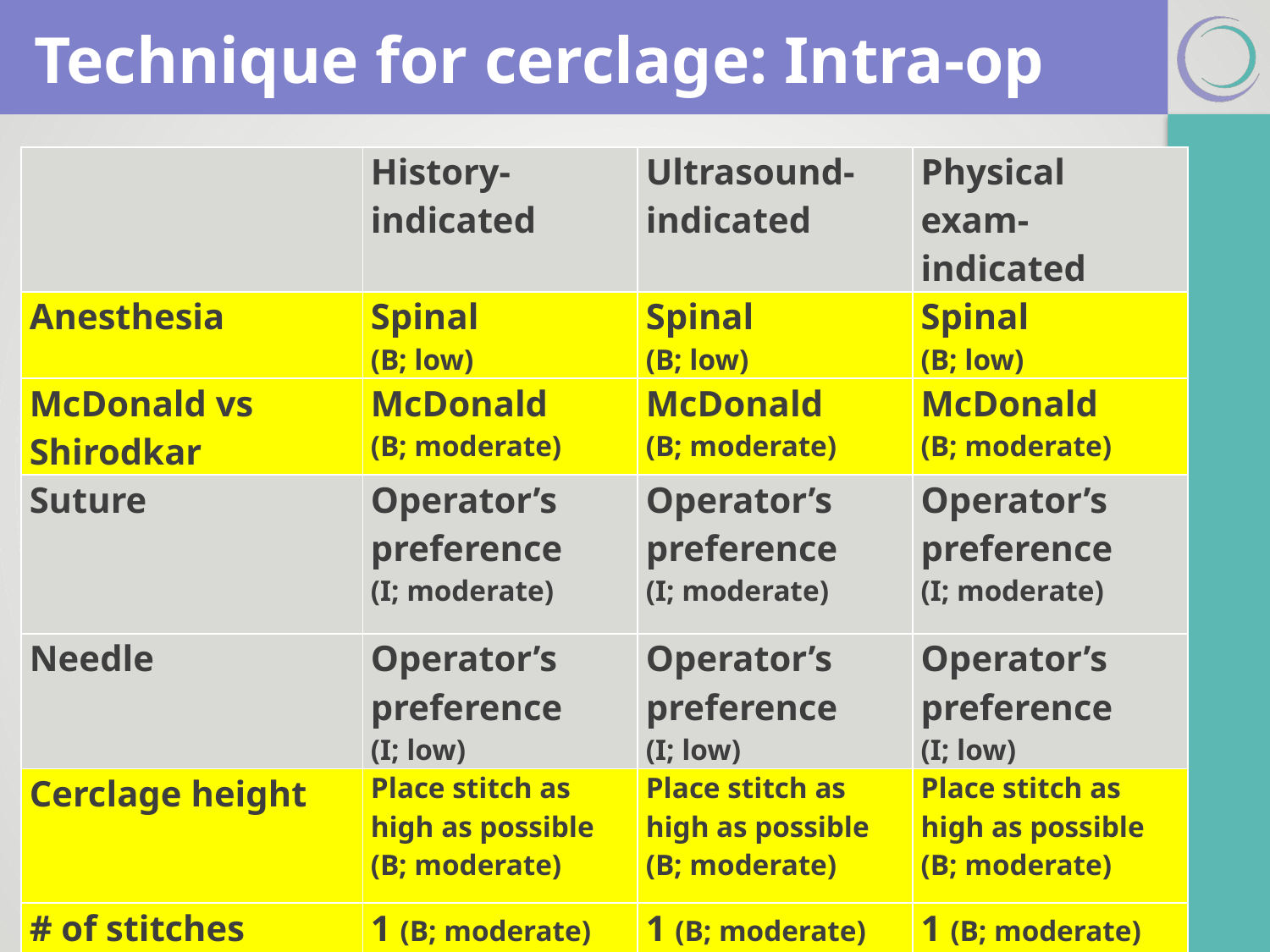

# Technique for cerclage: Intra-op
| | History-indicated | Ultrasound-indicated | Physical exam-indicated |
| --- | --- | --- | --- |
| Anesthesia | Spinal (B; low) | Spinal (B; low) | Spinal (B; low) |
| McDonald vs Shirodkar | McDonald (B; moderate) | McDonald (B; moderate) | McDonald (B; moderate) |
| Suture | Operator’s preference (I; moderate) | Operator’s preference (I; moderate) | Operator’s preference (I; moderate) |
| Needle | Operator’s preference (I; low) | Operator’s preference (I; low) | Operator’s preference (I; low) |
| Cerclage height | Place stitch as high as possible (B; moderate) | Place stitch as high as possible (B; moderate) | Place stitch as high as possible (B; moderate) |
| # of stitches | 1 (B; moderate) | 1 (B; moderate) | 1 (B; moderate) |

## Slide 8
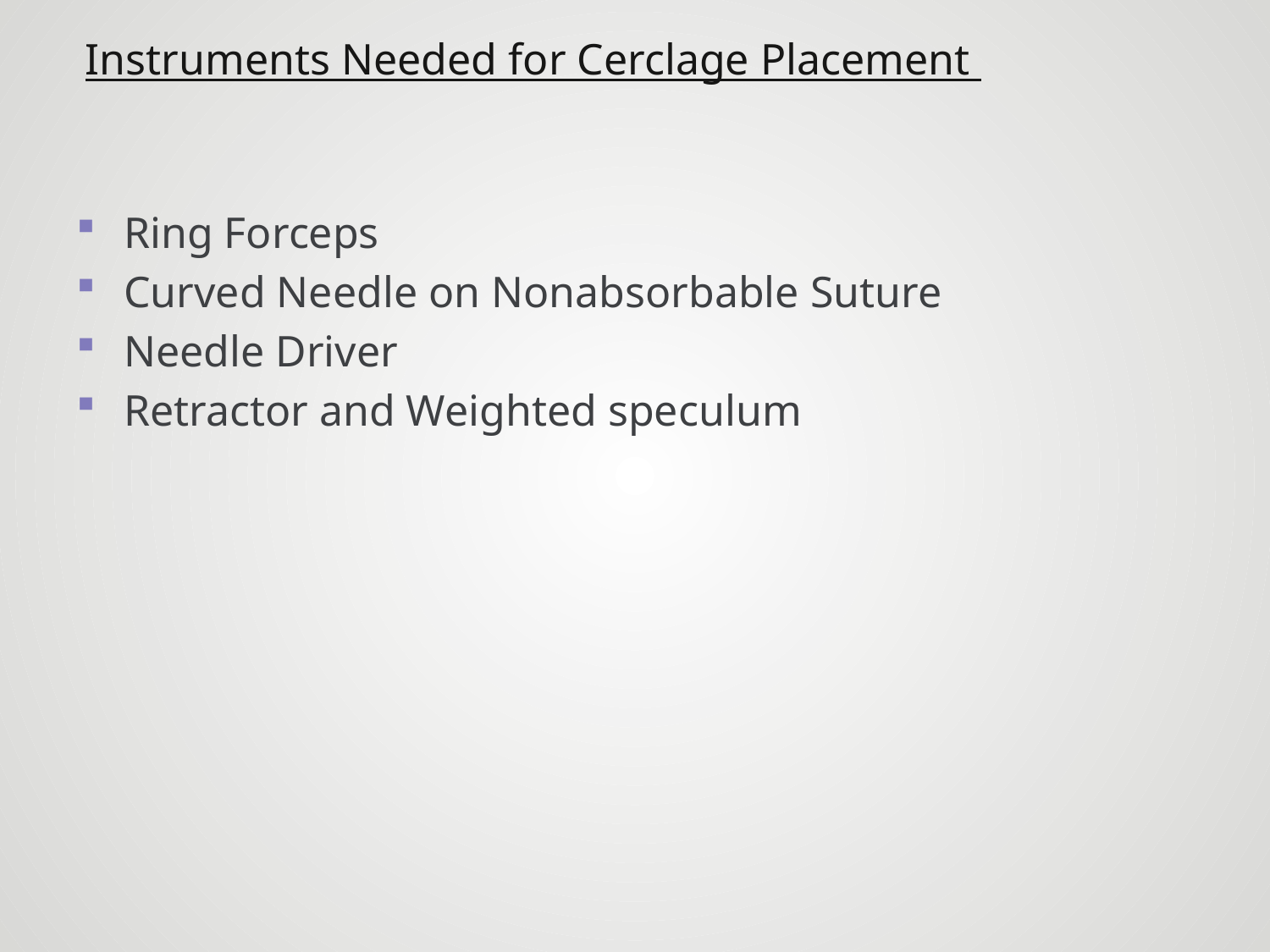

# Instruments Needed for Cerclage Placement
Ring Forceps
Curved Needle on Nonabsorbable Suture
Needle Driver
Retractor and Weighted speculum

## Slide 9
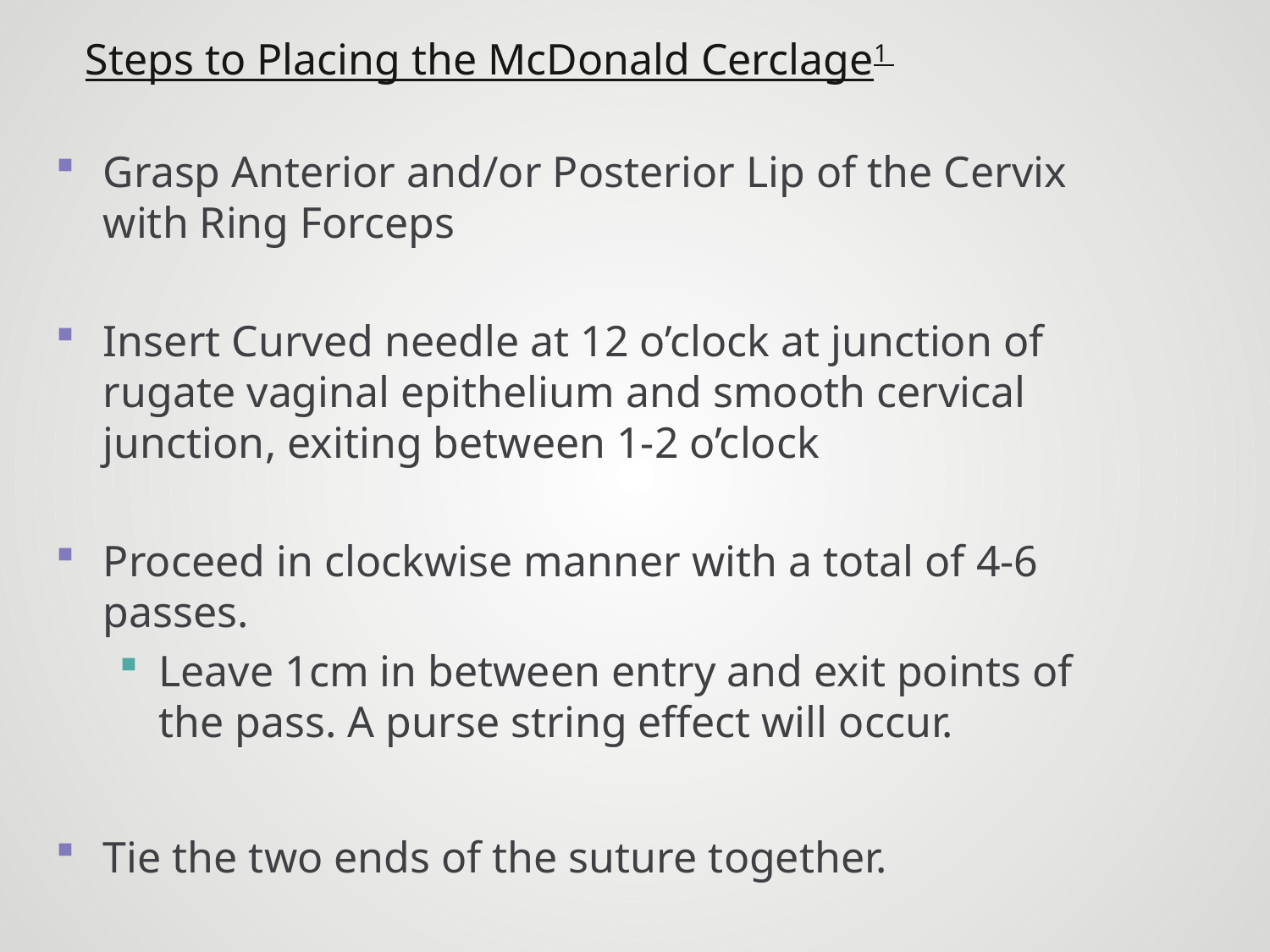

# Steps to Placing the McDonald Cerclage1
Grasp Anterior and/or Posterior Lip of the Cervix with Ring Forceps
Insert Curved needle at 12 o’clock at junction of rugate vaginal epithelium and smooth cervical junction, exiting between 1-2 o’clock
Proceed in clockwise manner with a total of 4-6 passes.
Leave 1cm in between entry and exit points of the pass. A purse string effect will occur.
Tie the two ends of the suture together.

## Slide 10
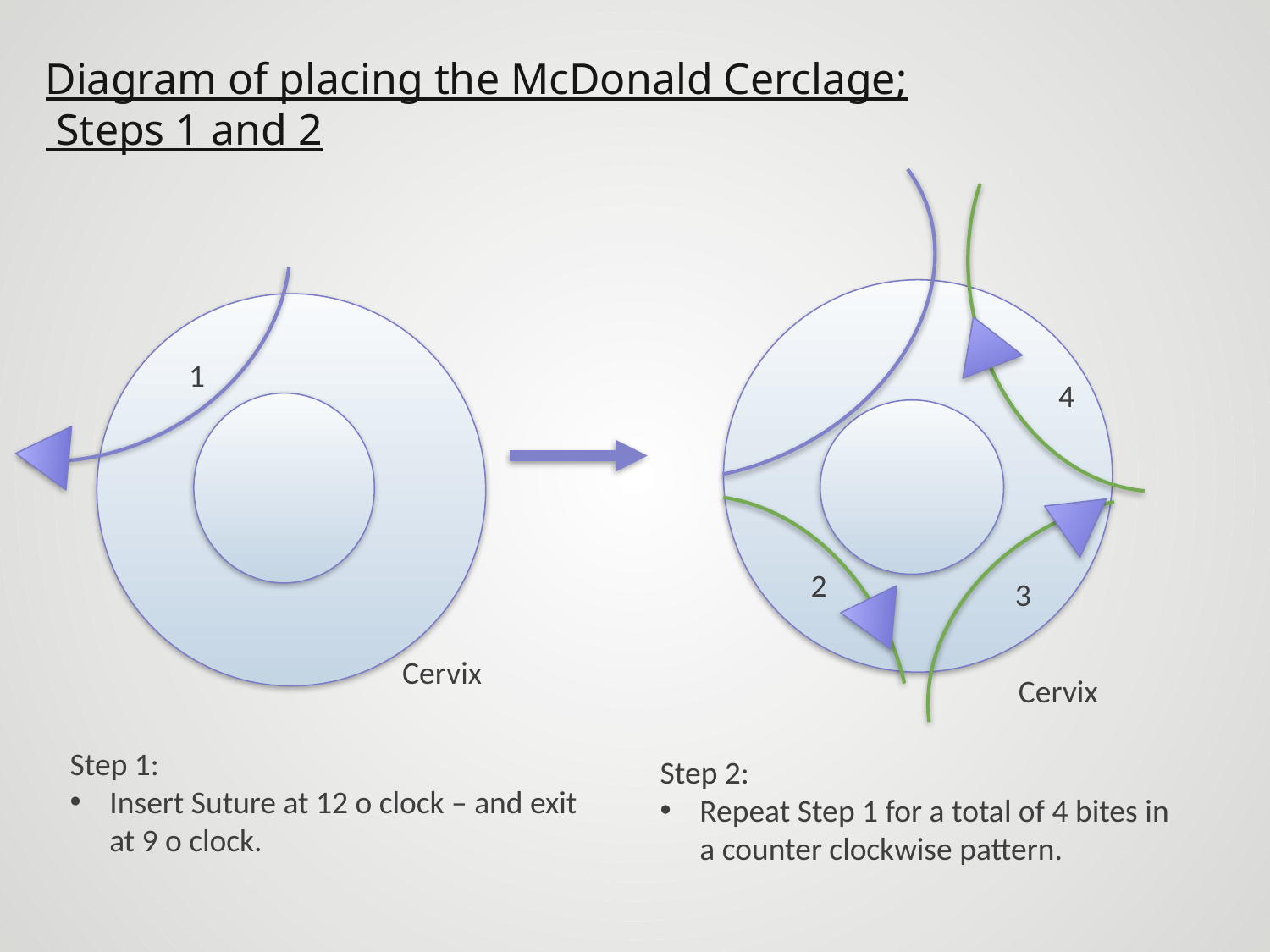

# Diagram of placing the McDonald Cerclage; Steps 1 and 2
1
4
2
3
Cervix
Cervix
Step 1:
Insert Suture at 12 o clock – and exit at 9 o clock.
Step 2:
Repeat Step 1 for a total of 4 bites in a counter clockwise pattern.

## Slide 11
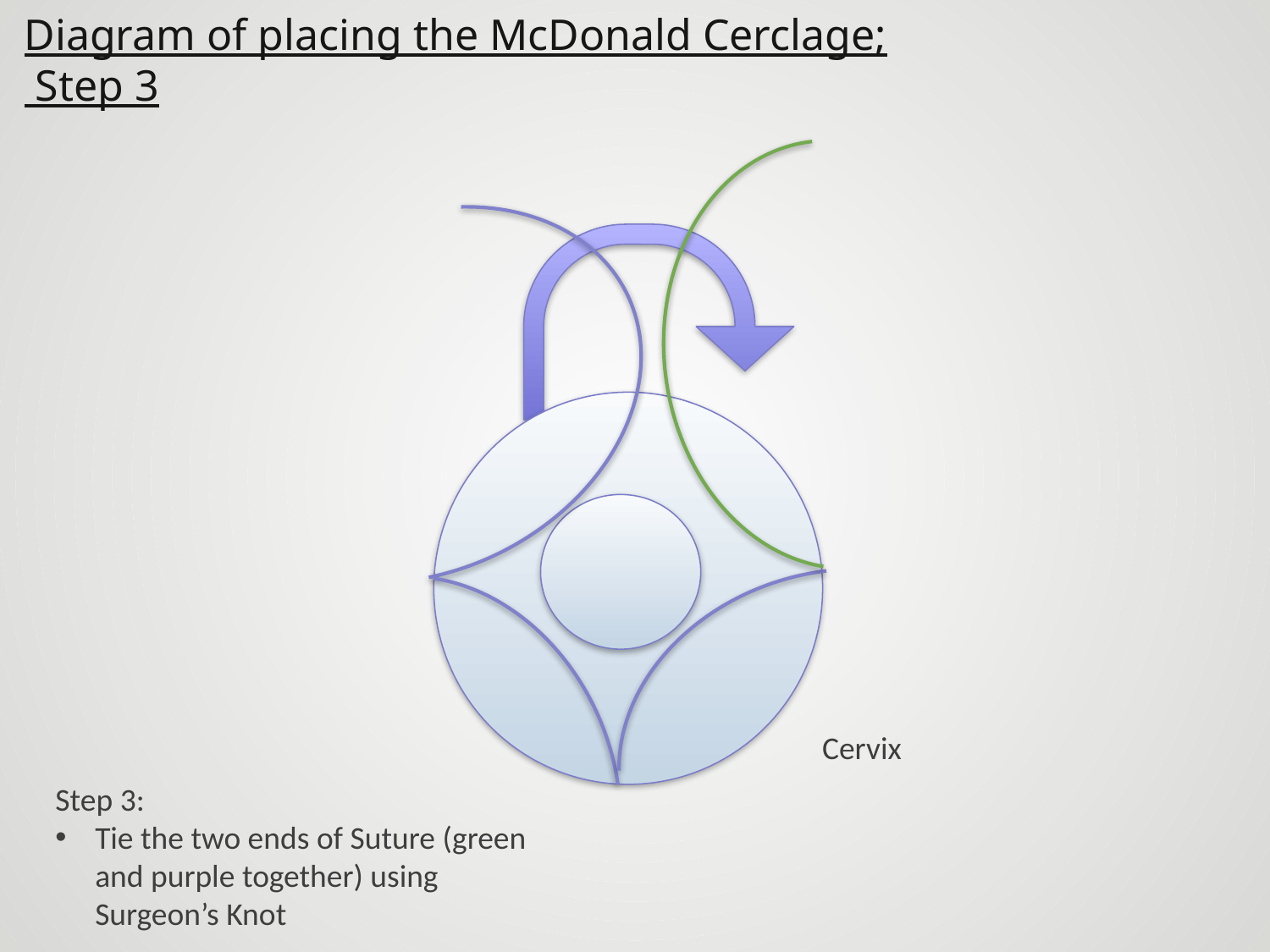

# Diagram of placing the McDonald Cerclage; Step 3
Cervix
Step 3:
Tie the two ends of Suture (green and purple together) using Surgeon’s Knot

## Slide 12
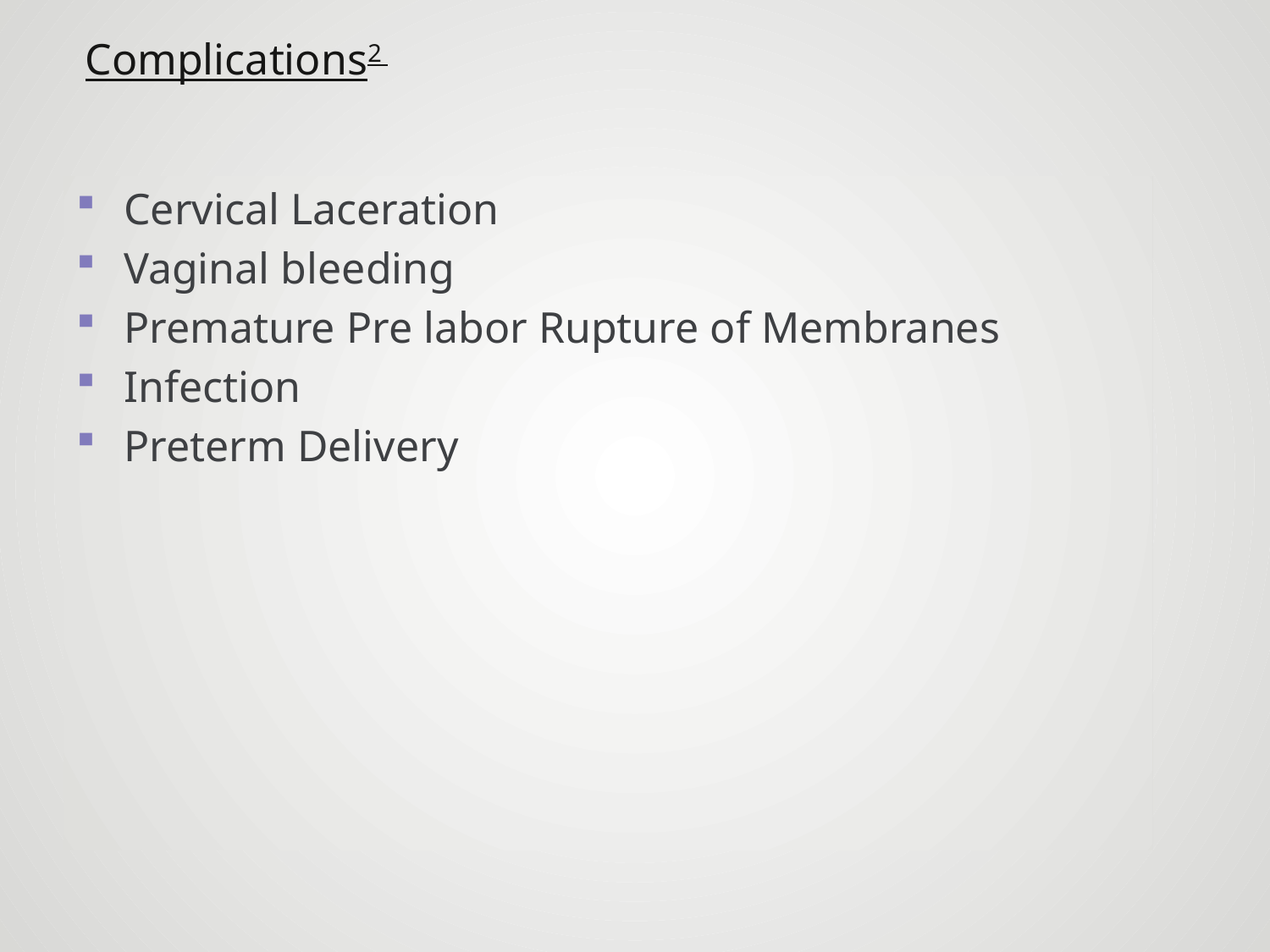

# Complications2
Cervical Laceration
Vaginal bleeding
Premature Pre labor Rupture of Membranes
Infection
Preterm Delivery

## Slide 13
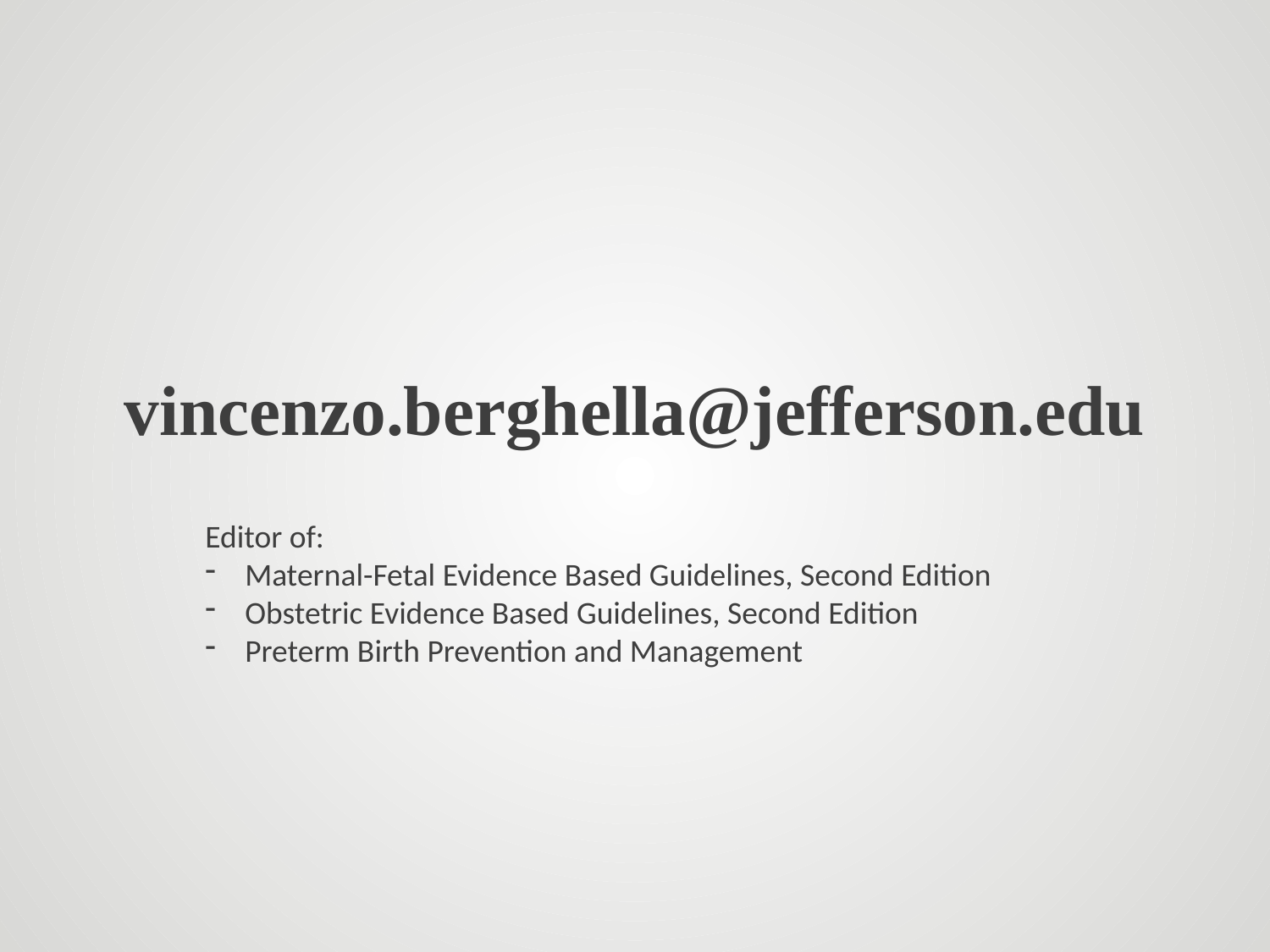

vincenzo.berghella@jefferson.edu
Editor of:
Maternal-Fetal Evidence Based Guidelines, Second Edition
Obstetric Evidence Based Guidelines, Second Edition
Preterm Birth Prevention and Management

## Slide 14
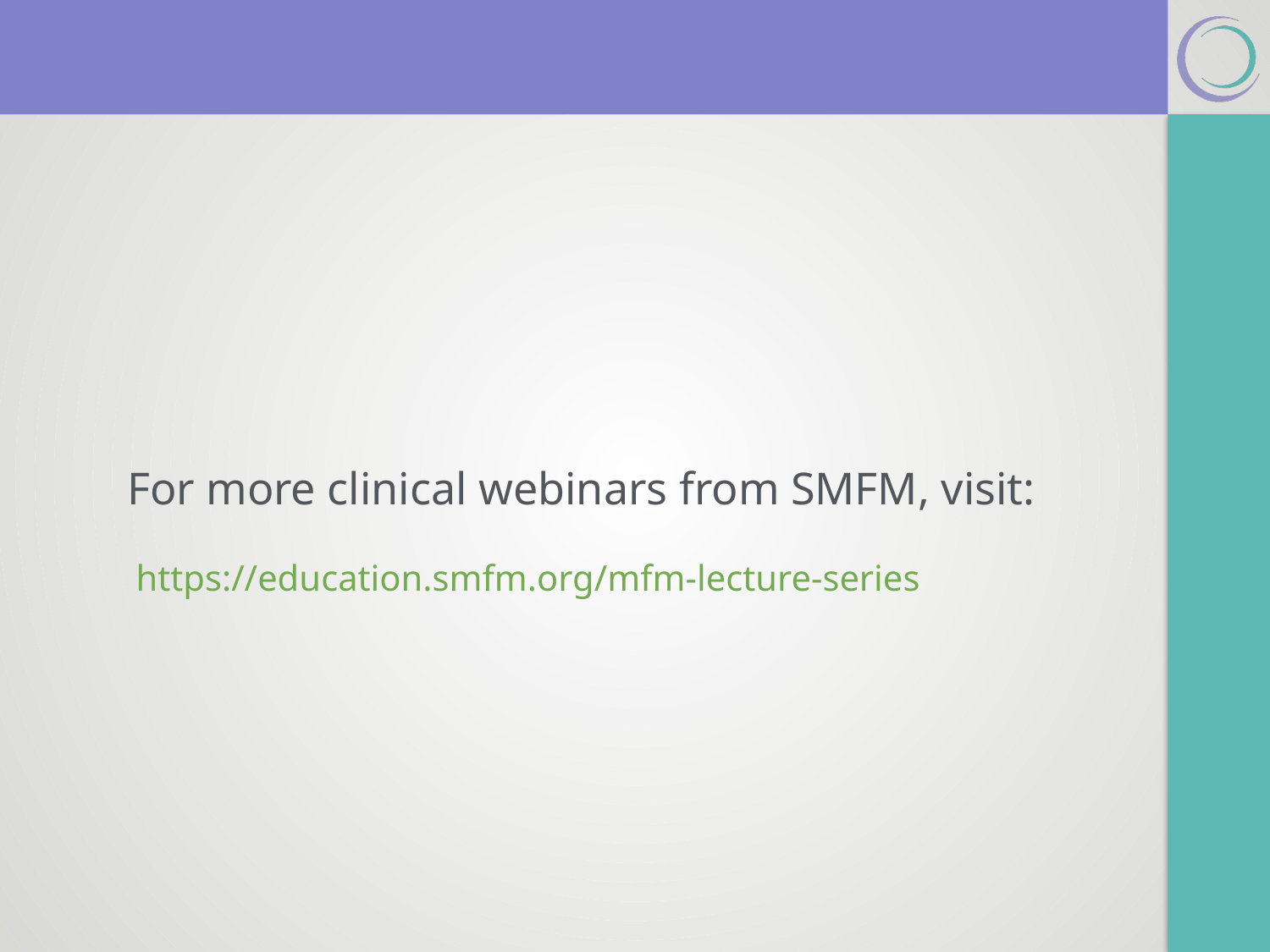

For more clinical webinars from SMFM, visit:
 https://education.smfm.org/mfm-lecture-series

## Slide 15
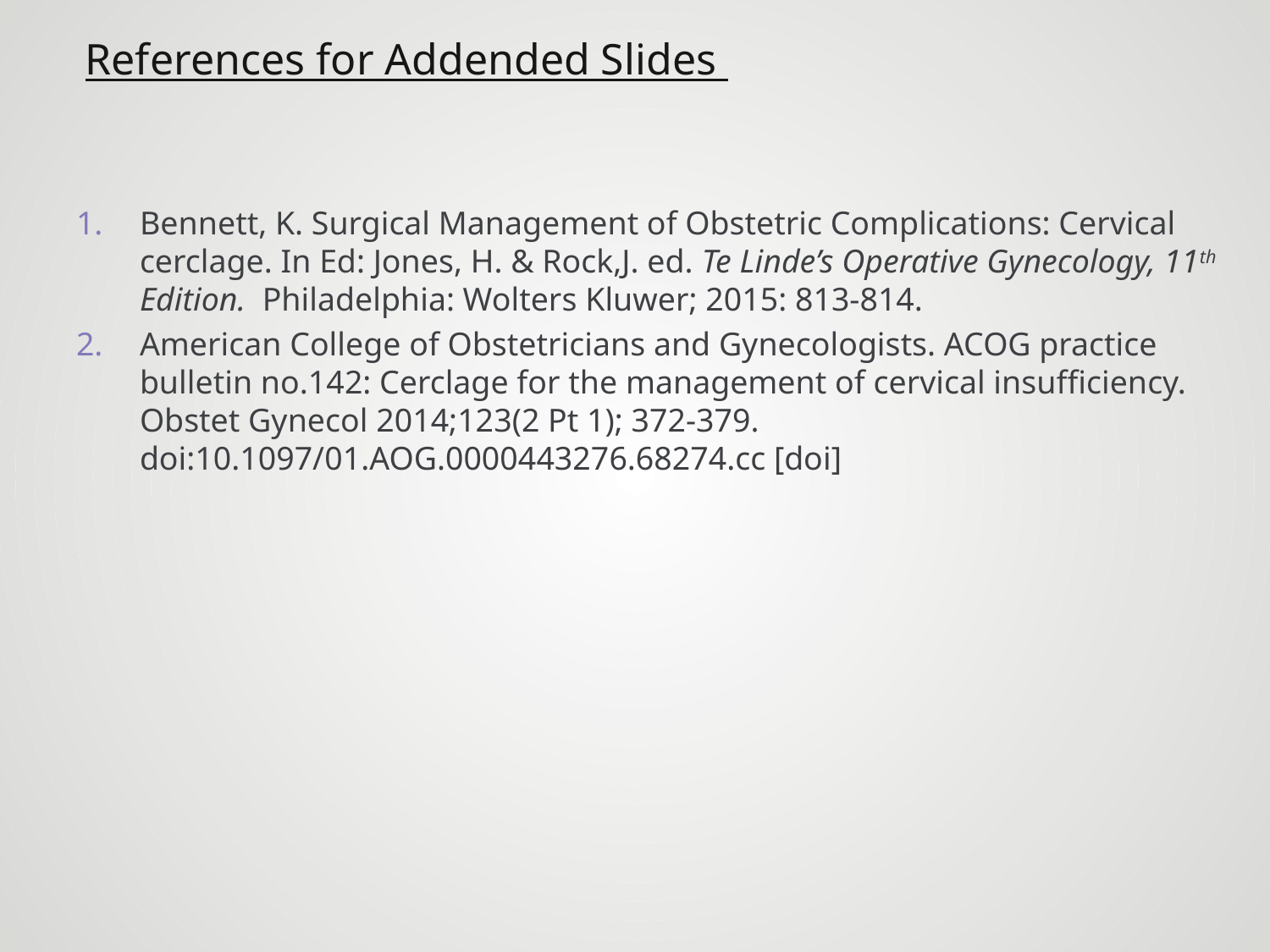

# References for Addended Slides
Bennett, K. Surgical Management of Obstetric Complications: Cervical cerclage. In Ed: Jones, H. & Rock,J. ed. Te Linde’s Operative Gynecology, 11th Edition. Philadelphia: Wolters Kluwer; 2015: 813-814.
American College of Obstetricians and Gynecologists. ACOG practice bulletin no.142: Cerclage for the management of cervical insufficiency. Obstet Gynecol 2014;123(2 Pt 1); 372-379. doi:10.1097/01.AOG.0000443276.68274.cc [doi]
