# Supplementary material for: A Novel Transvaginal Cervical Cerclage Model for Resident Training
Source: MedEdPORTAL. 2021 Mar 2;17:11102. doi: 10.15766/mep_2374-8265.11102 (PMC7970640; doi:10.15766/mep_2374-8265.11102)
Supplement: Supplementary file 1 — Cerclage Model Building Steps.docxAdapted Cervical Insufficiency Slide Deck.pptxPre- and Postsurvey.docxSkills Checklist.docx [file mep_2374-8265.11102-s001.zip › C. Pre- and Postsurvey.docx]

**Appendix C: Pre and Post Session Surveys**

**1. Pretest for Cerclage Model**

1. Age: __________ yrs.
2. Please indicate which gender you identify with: Male or Female
3. What year in residency are you? ___________
4. Have you completed a MFM rotation? _________ (yes/no)
5. Have you done a cerclage simulation before? ____________ (yes/no)
6. Have you performed a cerclage? _______________ (yes/no)
   1. If so, how many? ______________
7. I believe learning to perform a cerclage was easy.

| © | © | © | © | © | © |
| --- | --- | --- | --- | --- | --- |
| Strongly Disagree | Disagree | Neutral | Agree | Strongly  Agree | Not Applicable/No Prior Experience |

1. I am comfortable performing a cerclage.

| © | © | © | © | © |
| --- | --- | --- | --- | --- |
| Very Uncomfortable | Uncomfortable | Neutral | Comfortable | Very Comfortable |

1. At what level of supervision are you comfortable performing this procedure?*

| © | © | © | © | © |
| --- | --- | --- | --- | --- |
| Requires Supervision | Frequently Requires Supervision | Occasionally requires Supervision | Ready for Unsupervised Practice | Role Model |

1. I feel I need further training in cerclage placement.

| © | © | © | © | © |
| --- | --- | --- | --- | --- |
| Strongly Disagree | Disagree | Neutral | Agree | Strongly  Agree |

**2. Post-test for Cerclage Model**

1. I believe this training was helpful.

| © | © | © | © | © |
| --- | --- | --- | --- | --- |
| Strongly Disagree | Disagree | Neutral | Agree | Strongly  Agree |

1. Learning to perform a cerclage was easy using this simulation.

| © | © | © | © | © |
| --- | --- | --- | --- | --- |
| Strongly Disagree | Disagree | Neutral | Agree | Strongly  Agree |

1. I am comfortable performing cerclages following this simulation.

| © | © | © | © | © |
| --- | --- | --- | --- | --- |
| Very Uncomfortable | Uncomfortable | Neutral | Comfortable | Very Comfortable |

1. At what level of supervision are you comfortable performing this procedure now?*

| © | © | © | © | © |
| --- | --- | --- | --- | --- |
| Requires Supervision | Frequently Requires Supervision | Occasionally requires Supervision | Ready for Unsupervised Practice | Role Model |

1. I feel I need further training in cerclage placement.

| © | © | © | © | © |
| --- | --- | --- | --- | --- |
| Strongly Disagree | Disagree | Neutral | Agree | Strongly  Agree |

1. If I was the program director, I would recommend this training for future residents.

| © | © | © | © | © |
| --- | --- | --- | --- | --- |
| Strongly Disagree | Disagree | Neutral | Agree | Strongly  Agree |

*Questions 9 and 4 of Pre and Post- test, respectively, are adapted from the Accreditation Council for Graduate Medical Education. The obstetrics and gynecology milestone project. Published September 2015. Accessed October 18, 2018. And were used with permission.
