# Supplementary material for: A Novel Transvaginal Cervical Cerclage Model for Resident Training
Source: MedEdPORTAL. 2021 Mar 2;17:11102. doi: 10.15766/mep_2374-8265.11102 (PMC7970640; doi:10.15766/mep_2374-8265.11102)
Supplement: Supplementary file 1 — Cerclage Model Building Steps.docxAdapted Cervical Insufficiency Slide Deck.pptxPre- and Postsurvey.docxSkills Checklist.docx [file mep_2374-8265.11102-s001.zip › D. Skills Checklist.docx]

**Appendix D: Cerclage Placement Skills Checklist**

Name of Resident: _____________________________ Date:______________

Signature of Faculty Review: __________________________

| **Skill:** | **Competent in Skill:** | |
| --- | --- | --- |
| 1. Properly Visualize Cervix | Yes | No |
| 2. Call for proper instruments (needle driver, 0-vicryl suture) | Yes | No |
| 3. Load needle on needle driver | Yes | No |
| 4. Place stitch at 12 o clock with exit at 9 o’clock (or 3 o’clock if in clockwise direction) | Yes | No |
| 5. Complete 3 remaining stitches with final exit at 12 o’clock | Yes | No |
| 6. Properly place surgical knot at 12 o’clock | Yes | No |
